# (*E*)-Methyl 2-[(4-bromo-2-formyl­phen­oxy)meth­yl]-3-phenyl­acrylate

**DOI:** 10.1107/S1600536811047520

**Published:** 2011-11-16

**Authors:** T. Anuradha, G. Sivakumar, P. R. Seshadri, M. Bakthadoss

**Affiliations:** a Post Graduate & Research Department of Physics, Agurchand Manmull Jain College, Chennai 600 114, India; b Department of Organic Chemistry, University of Madras, Guindy Campus, Chennai 600 025, India

## Abstract

The C=C double bond in the title compound, C_18_H_15_BrO_4_, adopts an *E* configuration. The two rings are almost orthogonal to each other, making a dihedral angle of 82.8 (1)°. An intra­molecular C—H⋯O hydrogen bond occurs. The crystal structure is stabilized by inter­molecular C—H⋯O hydrogen bonds.

## Related literature

For background to the synthesis, see: Bakthadoss *et al.* (2009 ▶). For related phenyl acrylate compounds, see: Wang & Kong (2006 ▶); Wang *et al.* (2011 ▶). For the biological properties of cinnamate, see: Sharma (2011 ▶).
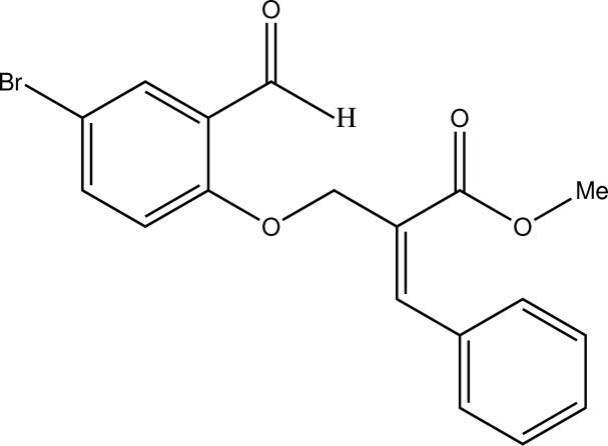


## Experimental

### 

#### Crystal data

C_18_H_15_BrO_4_
*M* *_r_* = 375.21
Monoclinic, 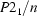

*a* = 8.2798 (2) Å
*b* = 22.1975 (5) Å
*c* = 9.2537 (2) Å
β = 99.857 (2)°
*V* = 1675.64 (7) Å^3^
*Z* = 4
Mo *K*α radiation
μ = 2.47 mm^−1^
*T* = 293 K
0.20 × 0.20 × 0.20 mm

#### Data collection

Bruker SMART APEXII area–detector diffractometer
16035 measured reflections
4185 independent reflections
2619 reflections with *I* > 2σ(*I*)
*R*_int_ = 0.033

#### Refinement

*R*[*F* ^2^ > 2σ(*F* ^2^)] = 0.042
*wR*(*F* ^2^) = 0.116
*S* = 0.99
4185 reflections
209 parameters
H-atom parameters constrained
Δρ_max_ = 0.68 e Å^−3^
Δρ_min_ = −0.53 e Å^−3^

### 

Data collection: *APEX2* (Bruker, 2008 ▶); cell refinement: *SAINT* (Bruker, 2008 ▶); data reduction: *SAINT*; program(s) used to solve structure: *SHELXS97* (Sheldrick, 2008 ▶); program(s) used to refine structure: *SHELXL97* (Sheldrick, 2008 ▶); molecular graphics: *ORTEP-3* (Farrugia, 1997 ▶); software used to prepare material for publication: *SHELXL97* and *PLATON* (Spek, 2009 ▶).

## Supplementary Material

Crystal structure: contains datablock(s) I, global. DOI: 10.1107/S1600536811047520/bt5689sup1.cif

Structure factors: contains datablock(s) I. DOI: 10.1107/S1600536811047520/bt5689Isup2.hkl

Supplementary material file. DOI: 10.1107/S1600536811047520/bt5689Isup3.cml

Additional supplementary materials:  crystallographic information; 3D view; checkCIF report

## Figures and Tables

**Table 1:** Hydrogen-bond geometry (Å, °)

*D*—H⋯*A*	*D*—H	H⋯*A*	*D*⋯*A*	*D*—H⋯*A*
C3—H3⋯O3	0.93	2.50	3.290 (3)	143
C2—H2⋯O1^i^	0.93	2.58	3.383 (4)	145
C13—H13⋯O1^ii^	0.93	2.55	3.291 (3)	137
C14—H14⋯O4^iii^	0.93	2.39	3.302 (4)	167

Symmetry codes: (i) 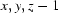; (ii) 
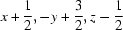
; (iii) 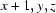.

**Fig. 1.**
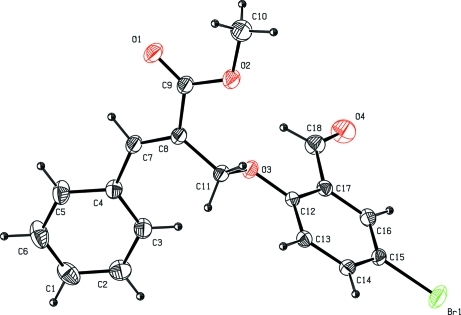
Molecular structure of the title compound, showing 30% probability displacement ellipsoids.

**Fig. 2.**
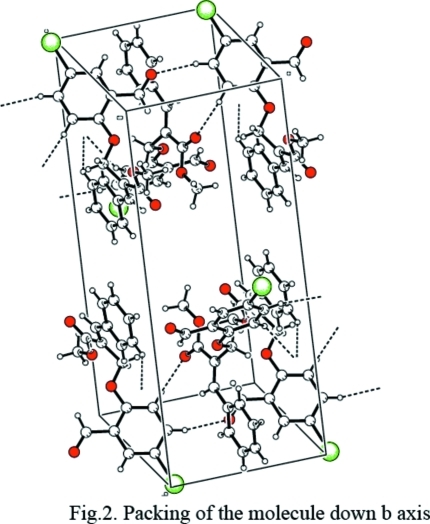
Packing of the molecule down b axis

## supplementary crystallographic information

### Comment

The title compound is used as precursor to obtain the desired tetra cyclic chromenopyran pyrimidinedione compounds *via* a tandem Knoevenagel intra-molecular hetero-Diels-Alder reaction (Bakthadoss *et al.*, 2009). Cinnamic acid and its derivatives including esters and carboxylic functional derivatives are used as important components in flavours, perfumes, synthetic indigo and pharmaceuticals. Cinnamate can act as optical filters or deactivate substrate molecules that have been excited by light for the protection polymers and organic substances. They are used as cosmetic grades and as sunscreen agents to reduce skin damage by blocking UV—A, B (Sharma, 2011). In view of this medicinal importance, the crystal structure determination of the title compound was carried out and the results are presented here.

The molecule adopts an E configuration about the C7 ═ C8 double bond. The dihedral angle between the best planes through the bromo-formylphenoxy group (C11—C18/O3/O4/Br) and phenylacrylate group (C1—C10/O1/O2) is 82.8 (1)°. The formyl group (C18/H18/04) is axial to the plane of the benzene ring to which it is attached as evidenced by the torsion angle C18—O4— C17— C12 of -7.9 (1)°.

From the bond length and bond angle analysis of the compound, the conformation of phenylacrylate group are comparable with corresponding values for the structure of ((E) – methyl 3-(3,4– dihydroxyphenyl)acrylate (Wang *et al.*, 2011).

The crystal packing is stabilized by intramolecular and intermolecular C—H···O hydrogen bonding interaction (Table 1).

### Experimental

A solution of 5-bromo-2-hydroxybenzaldehyde (1.0 mmol, 0.201 g) and potassium carbonate (2.0 mmol, 0.2293 g) in acetonitrile solvent (5 ml) was stirred for 15 minute at room temperature. To this solution, (*Z*)-methyl2-(bromomethyl)-3-phenylacrylate (1.2 mmol, 0.25 g) was added dropwise. After the completion of the reaction, as indicated by TLC, acetonitrile was evaporated. EtOAc (15 ml) and water (15 ml) were added to the crude mass. The organic layer was dried over anhydrous sodium sulfate. Removal of solvent led to the crude product, which was purified through pad of silica gel (100–200mesh) using ethylacetate and hexanes(1:9) as solvents. The pure title compound was obtained as a colourless solid (0.31 g, 83% yield). Recrystallization was carried out using ethylacetate as solvent.

### Refinement

All H atoms were positioned geometrically and allowed to ride on their parent atoms, with C—H = 0.93–0.97 Å and U_iso_(H) = 1.5*U*_eq_(C) for methyl H atoms and 1.2*U*_eq_(C) for other H atoms.

### Crystal data

**Table:**

C_18_H_15_BrO_4_	*F*(000) = 760
*M**_r_* = 375.21	*D*_x_ = 1.487 Mg m^−^^3^
Monoclinic, *P*2_1_/*n*	Mo *K*α radiation, λ = 0.71073 Å
Hall symbol: -P 2yn	Cell parameters from 4185 reflections
*a* = 8.2798 (2) Å	θ = 1.8–28.5°
*b* = 22.1975 (5) Å	µ = 2.47 mm^−^^1^
*c* = 9.2537 (2) Å	*T* = 293 K
β = 99.857 (2)°	Block, colourless
*V* = 1675.64 (7) Å^3^	0.20 × 0.20 × 0.20 mm
*Z* = 4	

### Data collection

**Table:**

Bruker SMART APEXII area–detector diffractometer	2619 reflections with *I* > 2σ(*I*)
Radiation source: fine-focus sealed tube	*R*_int_ = 0.033
graphite	θ_max_ = 28.5°, θ_min_ = 1.8°
ω and φ scans	*h* = −10→11
16035 measured reflections	*k* = −29→28
4185 independent reflections	*l* = −12→11

### Refinement

**Table:**

Refinement on *F*^2^	Primary atom site location: structure-invariant direct methods
Least-squares matrix: full	Secondary atom site location: difference Fourier map
*R*[*F*^2^ > 2σ(*F*^2^)] = 0.042	Hydrogen site location: inferred from neighbouring sites
*wR*(*F*^2^) = 0.116	H-atom parameters constrained
*S* = 0.99	*w* = 1/[σ^2^(*F*_o_^2^) + (0.0511*P*)^2^ + 0.7565*P*] where *P* = (*F*_o_^2^ + 2*F*_c_^2^)/3
4185 reflections	(Δ/σ)_max_ = 0.003
209 parameters	Δρ_max_ = 0.68 e Å^−^^3^
0 restraints	Δρ_min_ = −0.53 e Å^−^^3^

### Special details

**Table:**

Geometry. All e.s.d.'s (except the e.s.d. in the dihedral angle between two l.s. planes) are estimated using the full covariance matrix. The cell e.s.d.'s are taken into account individually in the estimation of e.s.d.'s in distances, angles and torsion angles; correlations between e.s.d.'s in cell parameters are only used when they are defined by crystal symmetry. An approximate (isotropic) treatment of cell e.s.d.'s is used for estimating e.s.d.'s involving l.s. planes.
Refinement. Refinement of *F*^2^ against ALL reflections. The weighted *R*-factor *wR* and goodness of fit *S* are based on *F*^2^, conventional *R*-factors *R* are based on *F*, with *F* set to zero for negative *F*^2^. The threshold expression of *F*^2^ > σ(*F*^2^) is used only for calculating *R*-factors(gt) *etc*. and is not relevant to the choice of reflections for refinement. *R*-factors based on *F*^2^ are statistically about twice as large as those based on *F*, and *R*- factors based on ALL data will be even larger.

### Fractional atomic coordinates and isotropic or equivalent isotropic displacement parameters (Å^2^)

**Table:**

	*x*	*y*	*z*	*U*_iso_*/*U*_eq_	
C1	−0.0256 (5)	0.60472 (17)	0.2844 (4)	0.0795 (10)	
H1	−0.0073	0.5836	0.2019	0.095*	
C2	−0.0193 (4)	0.66597 (16)	0.2866 (3)	0.0742 (9)	
H2	−0.0004	0.6868	0.2038	0.089*	
C3	−0.0407 (4)	0.69756 (13)	0.4103 (3)	0.0626 (7)	
H3	−0.0367	0.7394	0.4098	0.075*	
C4	−0.0678 (3)	0.66781 (12)	0.5351 (3)	0.0528 (6)	
C5	−0.0808 (5)	0.60533 (13)	0.5282 (3)	0.0779 (10)	
H5	−0.1045	0.5841	0.6087	0.093*	
C6	−0.0590 (5)	0.57457 (16)	0.4040 (4)	0.0914 (12)	
H6	−0.0671	0.5328	0.4017	0.110*	
C7	−0.0852 (4)	0.69664 (11)	0.6740 (3)	0.0541 (7)	
H7	−0.1372	0.6730	0.7353	0.065*	
C8	−0.0393 (3)	0.75109 (11)	0.7281 (3)	0.0488 (6)	
C9	−0.0742 (4)	0.76453 (12)	0.8777 (3)	0.0549 (7)	
C10	−0.0620 (6)	0.83736 (17)	1.0636 (3)	0.0921 (12)	
H10A	−0.1780	0.8396	1.0627	0.138*	
H10B	−0.0132	0.8760	1.0891	0.138*	
H10C	−0.0145	0.8080	1.1343	0.138*	
C11	0.0460 (3)	0.79772 (11)	0.6525 (3)	0.0478 (6)	
H11A	0.1082	0.8246	0.7238	0.057*	
H11B	0.1208	0.7788	0.5964	0.057*	
C12	−0.0297 (3)	0.87741 (10)	0.4790 (2)	0.0408 (5)	
C13	0.1324 (3)	0.88866 (11)	0.4670 (3)	0.0473 (6)	
H13	0.2153	0.8639	0.5147	0.057*	
C14	0.1704 (3)	0.93657 (11)	0.3844 (3)	0.0506 (6)	
H14	0.2791	0.9442	0.3769	0.061*	
C15	0.0484 (4)	0.97320 (10)	0.3130 (3)	0.0493 (6)	
C16	−0.1114 (3)	0.96307 (11)	0.3251 (3)	0.0494 (6)	
H16	−0.1930	0.9884	0.2774	0.059*	
C17	−0.1526 (3)	0.91524 (10)	0.4083 (3)	0.0435 (5)	
C18	−0.3232 (4)	0.90698 (15)	0.4253 (3)	0.0644 (8)	
H18	−0.3488	0.8737	0.4782	0.077*	
O1	−0.1327 (3)	0.73006 (10)	0.9529 (2)	0.0864 (8)	
O2	−0.0325 (3)	0.82006 (9)	0.9198 (2)	0.0700 (6)	
O3	−0.0789 (2)	0.83086 (7)	0.55632 (19)	0.0498 (4)	
O4	−0.4327 (3)	0.94054 (12)	0.3751 (3)	0.0897 (7)	
Br1	0.10355 (5)	1.038580 (15)	0.19991 (4)	0.08254 (17)	

### Atomic displacement parameters (Å^2^)

**Table:**

	*U*^11^	*U*^22^	*U*^33^	*U*^12^	*U*^13^	*U*^23^
C1	0.093 (3)	0.084 (2)	0.0561 (19)	0.0222 (19)	−0.0031 (16)	−0.0179 (17)
C2	0.090 (2)	0.083 (2)	0.0484 (17)	0.0013 (19)	0.0090 (15)	0.0005 (15)
C3	0.083 (2)	0.0534 (16)	0.0505 (15)	−0.0011 (14)	0.0078 (14)	−0.0005 (12)
C4	0.0624 (17)	0.0469 (14)	0.0469 (14)	−0.0033 (12)	0.0034 (12)	0.0009 (11)
C5	0.125 (3)	0.0500 (17)	0.0551 (18)	−0.0120 (17)	0.0043 (17)	−0.0015 (14)
C6	0.143 (4)	0.056 (2)	0.066 (2)	0.012 (2)	−0.010 (2)	−0.0116 (16)
C7	0.0684 (19)	0.0458 (14)	0.0495 (14)	−0.0071 (12)	0.0141 (12)	0.0047 (11)
C8	0.0566 (16)	0.0437 (13)	0.0476 (13)	−0.0035 (11)	0.0126 (11)	0.0045 (11)
C9	0.0700 (19)	0.0470 (15)	0.0491 (15)	−0.0082 (13)	0.0141 (13)	0.0016 (12)
C10	0.147 (4)	0.075 (2)	0.0601 (19)	−0.021 (2)	0.035 (2)	−0.0209 (17)
C11	0.0503 (16)	0.0440 (13)	0.0501 (14)	−0.0012 (11)	0.0114 (11)	0.0056 (11)
C12	0.0467 (15)	0.0335 (11)	0.0447 (13)	−0.0030 (10)	0.0150 (10)	−0.0039 (9)
C13	0.0451 (16)	0.0412 (13)	0.0571 (15)	0.0036 (11)	0.0128 (11)	0.0012 (11)
C14	0.0508 (16)	0.0453 (13)	0.0597 (16)	−0.0075 (12)	0.0210 (12)	−0.0054 (12)
C15	0.0695 (19)	0.0361 (12)	0.0449 (13)	−0.0085 (12)	0.0172 (12)	−0.0047 (10)
C16	0.0603 (18)	0.0421 (13)	0.0450 (14)	0.0045 (12)	0.0066 (11)	−0.0011 (11)
C17	0.0449 (15)	0.0411 (12)	0.0446 (13)	−0.0008 (10)	0.0083 (10)	−0.0050 (10)
C18	0.0521 (19)	0.0700 (19)	0.0709 (19)	−0.0001 (15)	0.0103 (14)	0.0036 (15)
O1	0.142 (2)	0.0641 (13)	0.0643 (13)	−0.0322 (13)	0.0482 (14)	−0.0047 (10)
O2	0.1062 (17)	0.0520 (11)	0.0564 (11)	−0.0188 (11)	0.0268 (11)	−0.0088 (9)
O3	0.0457 (10)	0.0423 (9)	0.0627 (11)	−0.0029 (7)	0.0129 (8)	0.0106 (8)
O4	0.0473 (13)	0.1048 (18)	0.115 (2)	0.0144 (13)	0.0078 (12)	0.0127 (16)
Br1	0.1225 (4)	0.0595 (2)	0.0689 (2)	−0.02273 (18)	0.0257 (2)	0.01451 (15)

### Geometric parameters (Å, °)

**Table:**

C1—C2	1.361 (5)	C10—H10B	0.9600
C1—C6	1.361 (5)	C10—H10C	0.9600
C1—H1	0.9300	C11—O3	1.445 (3)
C2—C3	1.380 (4)	C11—H11A	0.9700
C2—H2	0.9300	C11—H11B	0.9700
C3—C4	1.381 (4)	C12—O3	1.359 (3)
C3—H3	0.9300	C12—C13	1.389 (3)
C4—C5	1.392 (4)	C12—C17	1.393 (3)
C4—C7	1.466 (4)	C13—C14	1.377 (3)
C5—C6	1.375 (5)	C13—H13	0.9300
C5—H5	0.9300	C14—C15	1.374 (4)
C6—H6	0.9300	C14—H14	0.9300
C7—C8	1.338 (4)	C15—C16	1.366 (4)
C7—H7	0.9300	C15—Br1	1.890 (2)
C8—C9	1.492 (4)	C16—C17	1.388 (3)
C8—C11	1.493 (3)	C16—H16	0.9300
C9—O1	1.192 (3)	C17—C18	1.459 (4)
C9—O2	1.321 (3)	C18—O4	1.204 (4)
C10—O2	1.446 (3)	C18—H18	0.9300
C10—H10A	0.9600		
			
C2—C1—C6	119.4 (3)	H10A—C10—H10C	109.5
C2—C1—H1	120.3	H10B—C10—H10C	109.5
C6—C1—H1	120.3	O3—C11—C8	107.2 (2)
C1—C2—C3	120.7 (3)	O3—C11—H11A	110.3
C1—C2—H2	119.7	C8—C11—H11A	110.3
C3—C2—H2	119.7	O3—C11—H11B	110.3
C2—C3—C4	120.9 (3)	C8—C11—H11B	110.3
C2—C3—H3	119.6	H11A—C11—H11B	108.5
C4—C3—H3	119.6	O3—C12—C13	124.1 (2)
C3—C4—C5	117.4 (3)	O3—C12—C17	116.4 (2)
C3—C4—C7	125.4 (2)	C13—C12—C17	119.5 (2)
C5—C4—C7	117.2 (2)	C14—C13—C12	119.9 (2)
C6—C5—C4	120.9 (3)	C14—C13—H13	120.0
C6—C5—H5	119.6	C12—C13—H13	120.0
C4—C5—H5	119.6	C15—C14—C13	120.3 (2)
C1—C6—C5	120.6 (3)	C15—C14—H14	119.8
C1—C6—H6	119.7	C13—C14—H14	119.9
C5—C6—H6	119.7	C16—C15—C14	120.4 (2)
C8—C7—C4	131.1 (2)	C16—C15—Br1	120.1 (2)
C8—C7—H7	114.4	C14—C15—Br1	119.5 (2)
C4—C7—H7	114.4	C15—C16—C17	120.3 (2)
C7—C8—C9	116.1 (2)	C15—C16—H16	119.8
C7—C8—C11	125.3 (2)	C17—C16—H16	119.8
C9—C8—C11	118.6 (2)	C16—C17—C12	119.5 (2)
O1—C9—O2	122.6 (2)	C16—C17—C18	119.4 (2)
O1—C9—C8	125.2 (2)	C12—C17—C18	121.1 (2)
O2—C9—C8	112.2 (2)	O4—C18—C17	124.1 (3)
O2—C10—H10A	109.5	O4—C18—H18	118.0
O2—C10—H10B	109.5	C17—C18—H18	118.0
H10A—C10—H10B	109.5	C9—O2—C10	116.2 (2)
O2—C10—H10C	109.5	C12—O3—C11	117.67 (18)

### Hydrogen-bond geometry (Å, °)

**Table:**

*D*—H···*A*	*D*—H	H···*A*	*D*···*A*	*D*—H···*A*
C3—H3···O3	0.93	2.50	3.290 (3)	143.
C7—H7···O1	0.93	2.37	2.777 (3)	106.
C11—H11A···O2	0.97	2.32	2.708 (3)	103.
C18—H18···O3	0.93	2.42	2.752 (4)	101.
C2—H2···O1^i^	0.93	2.58	3.383 (4)	145.
C13—H13···O1^ii^	0.93	2.55	3.291 (3)	137.
C14—H14···O4^iii^	0.93	2.39	3.302 (4)	167.

Symmetry codes: (i) *x*, *y*, *z*−1; (ii) *x*+1/2, −*y*+3/2, *z*−1/2; (iii) *x*+1, *y*, *z*.
